# Communication strategies for adults in palliative care: the speech-language therapists’ perspective

**DOI:** 10.1186/s12904-024-01382-x

**Published:** 2024-02-21

**Authors:** Cátia Dias, Inês Tello Rodrigues, Hernâni Gonçalves, Ivone Duarte

**Affiliations:** 1 Department of Community Medicine, Information and Health Decision Sciences (MEDCIDS), Faculty of Medicine, University of Porto, Porto, Portugal; 2 Centre for Innovative Care and Health Technology (ciTechcare), Polytechnic of Leiria, Leiria, Portugal; 3 Alcoitão School of Health Sciences (ESSAlcoitão), Alcabideche, Portugal; 4 Centre for Health Technology and Services Research (CINTESIS@RISE), Faculty of Medicine, University of Porto, Porto, Portugal

**Keywords:** Communication, Palliative care, Speech therapy, Adult

## Abstract

**Background:**

Communication disorders are a challenge that many patients in palliative care (PC) may encounter. This intervention area is emerging for the speech-language therapist (SLT), the professional who works in preventing, assessing, diagnosing, and treating human communication disorders. This study aims to identify and classify the communication strategies considered most important by SLTs for use in PC and evaluate whether there are any differences in perception regarding the importance of strategies between SLTs with and without PC experience.

**Methods:**

This cross-sectional quantitative study was conducted using a survey, which employed a well-structured, self-completion questionnaire previously validated by a panel of experts with over six years of PC experience.

**Results:**

The strategies rated as most important within each group were the following: (i) adjust the patient’s position and minimise environmental noise; (ii) establish eye contact and adjust the pace of speech; (iii) adjust the language level and raise one topic at a time; (iv) use images of the patient’s interests and their personal objects; (v) use orality and multimodal form; (vi) use simplified language and structured pauses; and (vii) use tables with images and books with pictures.

**Conclusions:**

Verbal and non-verbal strategies were rated as highly important. There was no evidence of differences in perception in terms of importance between the SLTs with or without experience in PC, but more studies are needed to support this aspect. The patient’s communication ability is one of the cornerstones of PC quality. Through their actions, speech-language professionals could empower the patient with strategies so that they can autonomously and self-determinedly express their experiences and most significant needs.

**Supplementary Information:**

The online version contains supplementary material available at 10.1186/s12904-024-01382-x.

## Background

Palliative care (PC) is person-centred care aimed at relieving pain and symptoms resulting from the advanced stage of a disease that cannot be cured [1]. This approach focuses on promoting the quality of life of families and patients, helping them to face all the challenges associated with a terminal illness [1]. In addition, advocating symptom control, PC also includes support for the person’s psychological, spiritual, and social suffering [1]. In this sense, a communication disorder could be a significant barrier to implementing the advocated underlying objectives [2].

In the advanced stages of an illness, communication is often very compromised [3]. According to the literature, in PC, many patients are expected to have disturbances in both communication and swallowing due to the characteristics and progression of their health conditions [2, 4]. Pollens [2] refers to O’Reilly and Walshe’s research of 2015 [5] (involving 322 speech therapists working in PC in the Republic of Ireland, the United Kingdom, the United States, Canada, Australia, and New Zealand), centred on speech-language therapists (SLTs) in PC at an international level [2, 5]. Cases of advanced dementia, neurodegenerative diseases, and tumours are the group of pathologies that SLTs most frequently accompany, significantly impacting communicative competence [2].

Following this line of thought, a study was conducted in Portugal with 38 adult PC patients and 26 family members/informal caregivers [6]. The study concluded that 55.3% of patients reported having difficulty communicating. The option “communicate with difficulty” was selected by 34.2% of the patients. In turn, 57.7% of families and/or informal caregivers refer to this difficulty as primordial, and the highest percentage for this group (30.8%) was also related to the option “communicate with difficulty” [6].

The patient’s communication ability is one of the pillars of end-of-life quality care [2]. In the face of a communication disorder, patients may be limited in their ability to communicate their needs, concerns, and decisions verbally, and their inclusion in decision-making is also often overlooked and their views misinterpreted [2]. According to the American Speech-Language-Hearing Association (2016), the SLT should prevent, assess, diagnose, and treat human communication disorders. Pollens [2] particularises the work of the SLT in PC: (i) promote competencies in the patients so that they can communicate their needs (e.g., healthcare, emotional, and spiritual needs) autonomously; (ii) promote decision-making and fulfilment of end-of-life objectives; (iii) create competencies in the team so that they can discuss issues related to death with the patient; and (iv) encourage social and emotional closeness between patients and their families [2].

Other studies emphasise that communication barriers limit the patients’ participation in their care plan [2, 7–9]. As a result of this concern, strategies have been developed to enhance the communicative functionality of the person in a clinical care setting [7]. For example, Stead and McDonnell [10] created communicative resources aimed at people with aphasia to support the realisation of their advance directives [2, 10].

Another study on people with dementia showed that using tables with images (including their respective captions) allowed users to communicate their preferences to the nursing team regarding the healthcare they wanted to receive [11]. Furthermore, communication strategies (also known as compensatory strategies) allow the person to interact with communication partners, bypassing the barriers imposed by the disorder’s characteristics [9]. In the face of these difficulties, verbal, non-verbal, and visual strategies, assistive technologies, environmental strategies (physical space), formal strategies, and various forms of communication can be used [11–13].

These strategies are a new expanded communicative behaviour, often acquired spontaneously and used systematically to overcome communication barriers [9]. The implementation of communication strategies is one of the competencies of SLTs, materialising in a specific way by using materials, symbols, and/or techniques, significantly promoting the patients’ participation in the final phase of their lives [2, 9, 14]. However, the research related to the existence of standardised protocols and procedures is scarce, and more knowledge is needed.

It is important to point out that the literature recognises the need for communication skills to support the quality of life of patients with advanced illnesses and their families [2, 15]. Moreover, it is fundamental for patients to be able to choose freely and autonomously, communicate decisions and needs, and express what they do and do not want and what they don’t want for themselves while receiving PC [2, 15].

Currently, no comprehensive summary exists for the communication strategies mentioned in the literature to support PC patients. The roles of SLTs in communication with PC patients are very relevant, but further research is needed. Given these gaps, this study sought to: (a) understand the SLTs perception of the most important communication strategies to implement with adults in a PC context; and (b) understand if there are differences in perception, and the attribution of importance to the strategies, between professionals who have PC experience and those who do not.

## Methods

This cross-sectional quantitative study involved 97 SLTs: 40 with PC experience and 57 without experience in this area. Data was collected using a self-completion questionnaire structured with closed-ended questions available online (in Portuguese). The data collection instrument was developed for research purposes, as there were no questionnaires on this subject, and then validated by a panel of experts—the Delphi panel (supplemental file 1). The criteria for inclusion in this panel included the following aspects: (i) four clinicians with more than six years of professional experience; (ii) experience with adults in a PC situation; and (iii) experience in the field of communication in PC. The Delphi technique was implemented in two phases to validate the content of the data collection instrument.

The final questionnaire was organised as follows: section i) socio-demographic information (gender; age; years of service; place of work; age groups the clinician works with; experience with the adult PC population; and section ii) communication strategies included in seven categories: (1) environmental strategies, (2) non-verbal strategies, (3) verbal strategies, (4) visual strategies, (5) communication forms, (6) formal strategies, and (7) assistive technologies. A Lickert-type response scale was used with the following designations: 1 - “*not important*”, 2 - “*not very important*”, 3 - “*important*”, 4 - “*very important*”.

Participants were recruited using the snowball sampling technique. The inclusion criteria for taking part in the study was Portuguese speech therapists working in Portugal. The study complied with the ethical principles set out in the Declaration of Helsinki (2013), and the research was approved by the Ethics Committee of the University Hospital Centre of São João (reference no. 60 − 19). Those responsible for the study agreed to the consent form, and all participants gave their free and informed consent in the online questionnaire form.

The data was exported from Google® Forms to Microsoft Excel® 2016, and all statistical analyses were carried out in SPSS® Statistics (version 25, IBM Corp., Armonk, NY, USA) and RStudio (version 2023.9.1.494). In the descriptive analysis of the results, categorical variables were described using absolute and relative frequencies, while the continuous variable was described using the median, minimum and maximum. The Friedman test was used to compare the strategies within each category, and the mean rank was used to identify the two strategies that were given the greatest importance within each category for the purposes of analysis. The chi-square or Fisher’s test was used when appropriate to compare the groups of SLTs. The significance level was set at 0.05.

## Results

Participants in the study ranged in age from 22 to 57, mostly female (90%), and most of them had up to five years of professional experience and worked in long-term care, home care, and clinics. Regarding the population with which they work (there may be more than one at the same time), 86% of therapists work with paediatric, 67% with adult, and 61% with youth populations (Table 1). As shown in Table 1, SLTs with PC experience work more in long-term care (*p* < 0.001) and nursing homes (*p* = 0.005) and less in schools (*p* = 0.001), as well as working more with adults (*p* < 0.001) and less with children (*p* < 0.001).

**Fig. 1:**
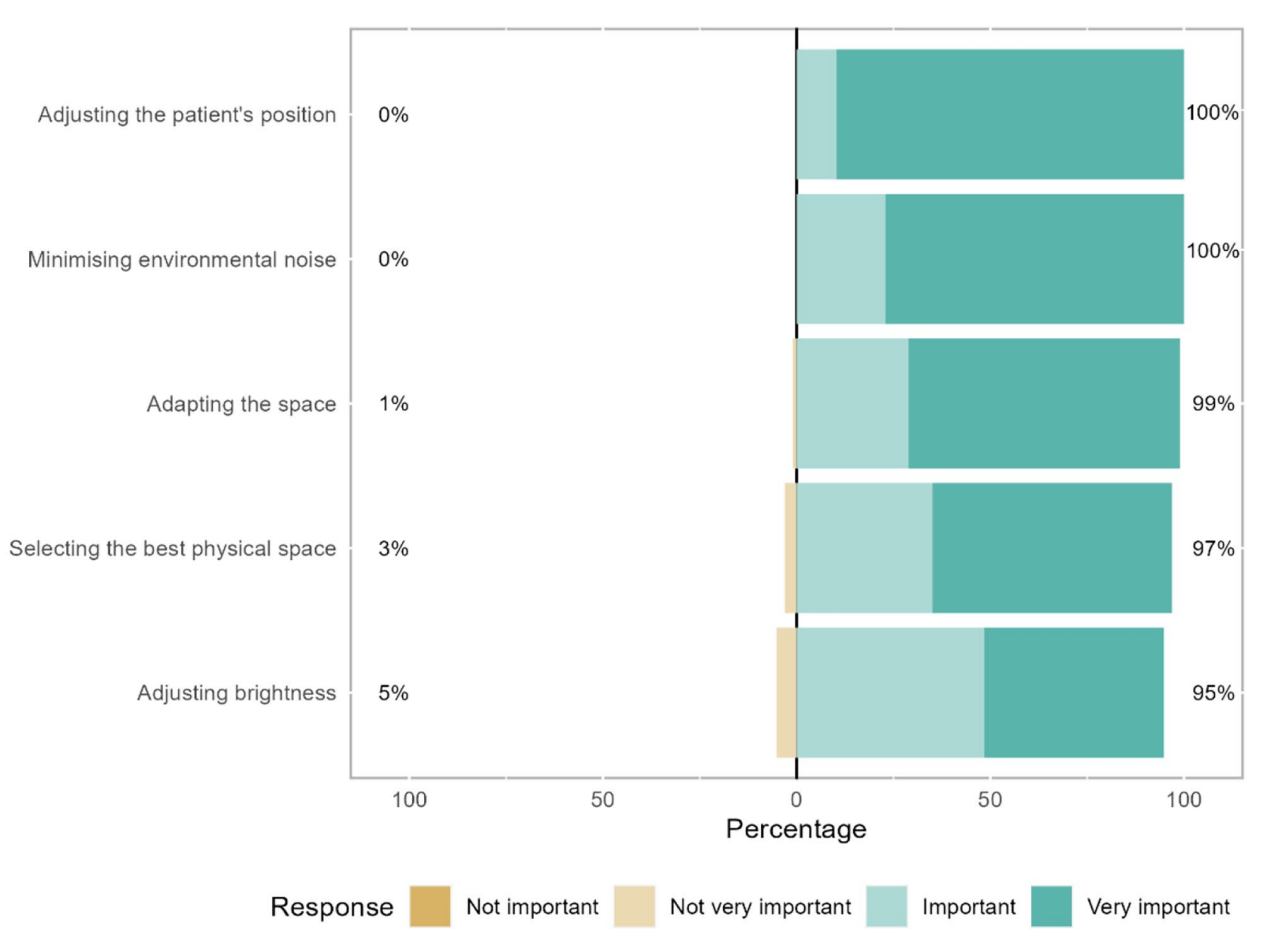
Environmental communication strategies

There were statistically significant differences between the strategies within each of the seven categories, each with *p* < 0.001. Within the environmental strategies group, 90% of therapists find adjusting the patient’s position (mean rank = 3.54) “very important”, while the remaining therapists consider it “important”. Minimising environmental noise (mean rank = 3.22) is considered by 77% of therapists to be “very important”, while the remaining consider it “important” (Fig. 1; Table 2).

**Fig. 2:**
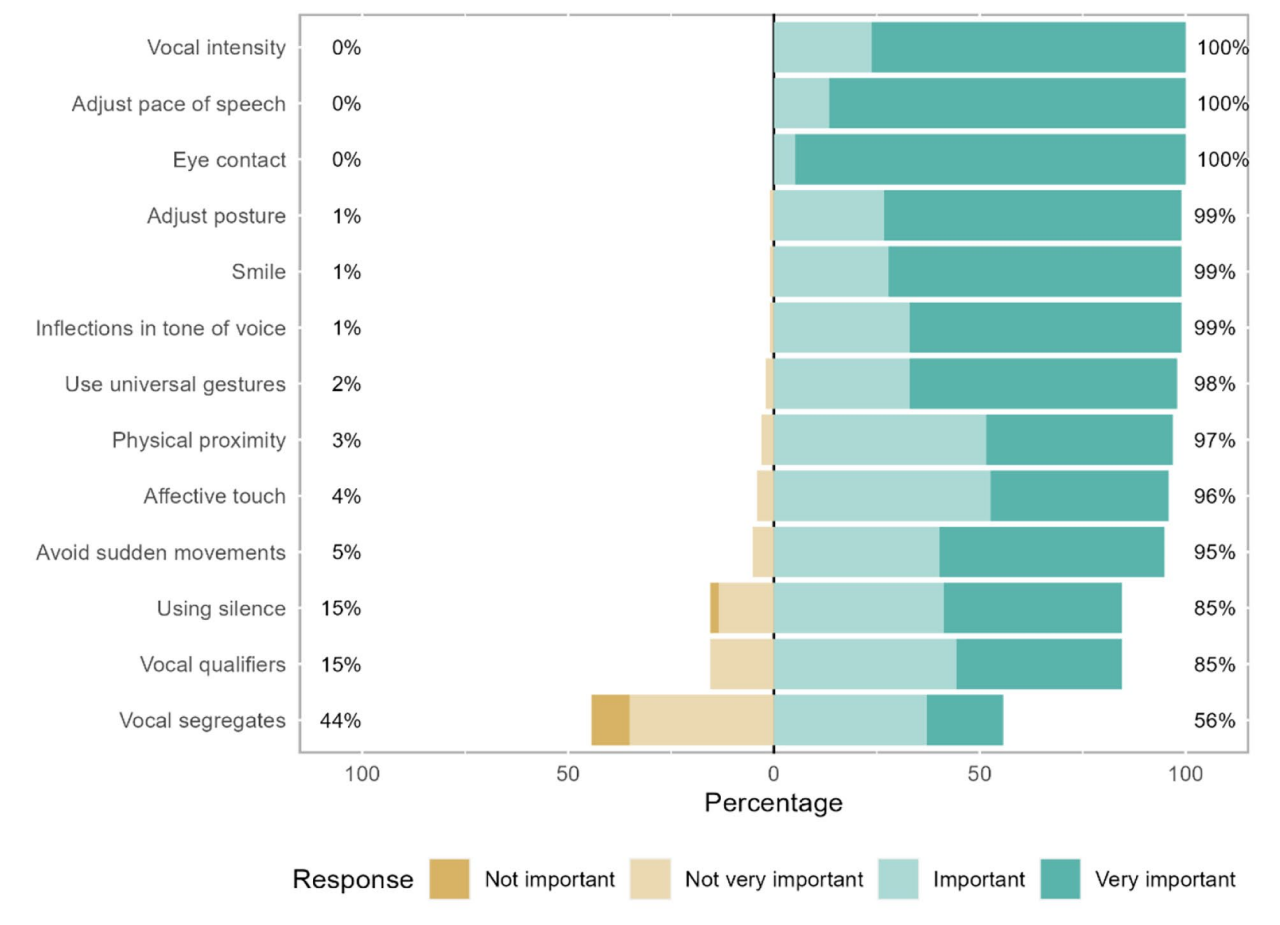
Non-verbal communication strategies

In terms of non-verbal strategies, eye contact is “very important” for 95% of the participants and “important” for the remaining participants. The pace of speech (mean rank = 8.89) is considered “very important” for 87% of participants and “important” for the remaining participants (Fig. 2; Table 2).

**Fig. 3:**
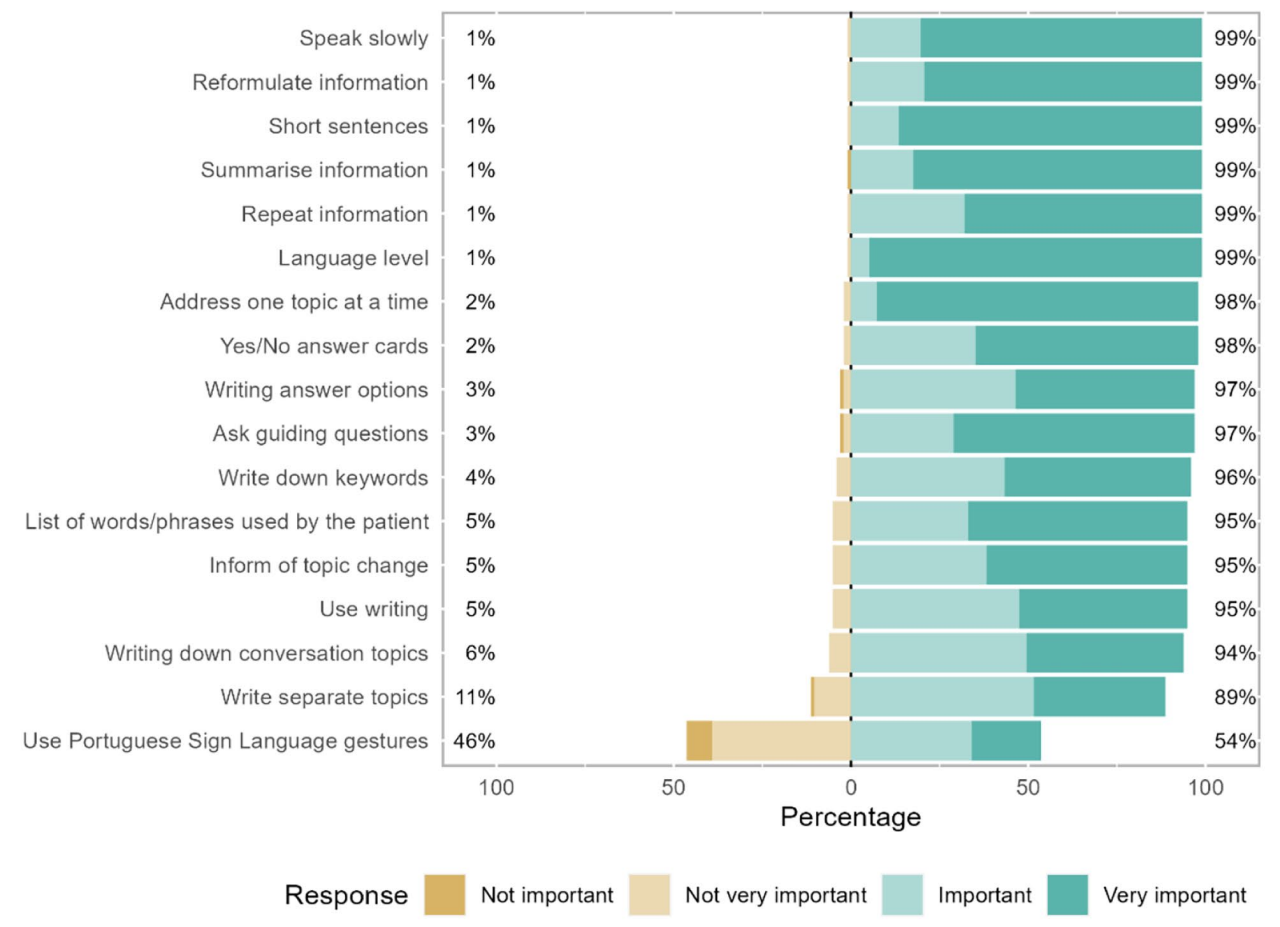
Verbal communication strategies

Regarding verbal strategies, linguistic adjustment is most prominent (mean rank = 11.61), as 94% of therapists responded that it was “very important”. In contrast, the remaining therapists answered that it was “important” or “not very important”. Addressing one topic at a time (mean rank = 11.32) is considered by 91% of respondents to be “very important”, while the remaining respondents consider it to be “important” or “not very important” (Fig. 3; Table 2).

**Fig. 4:**
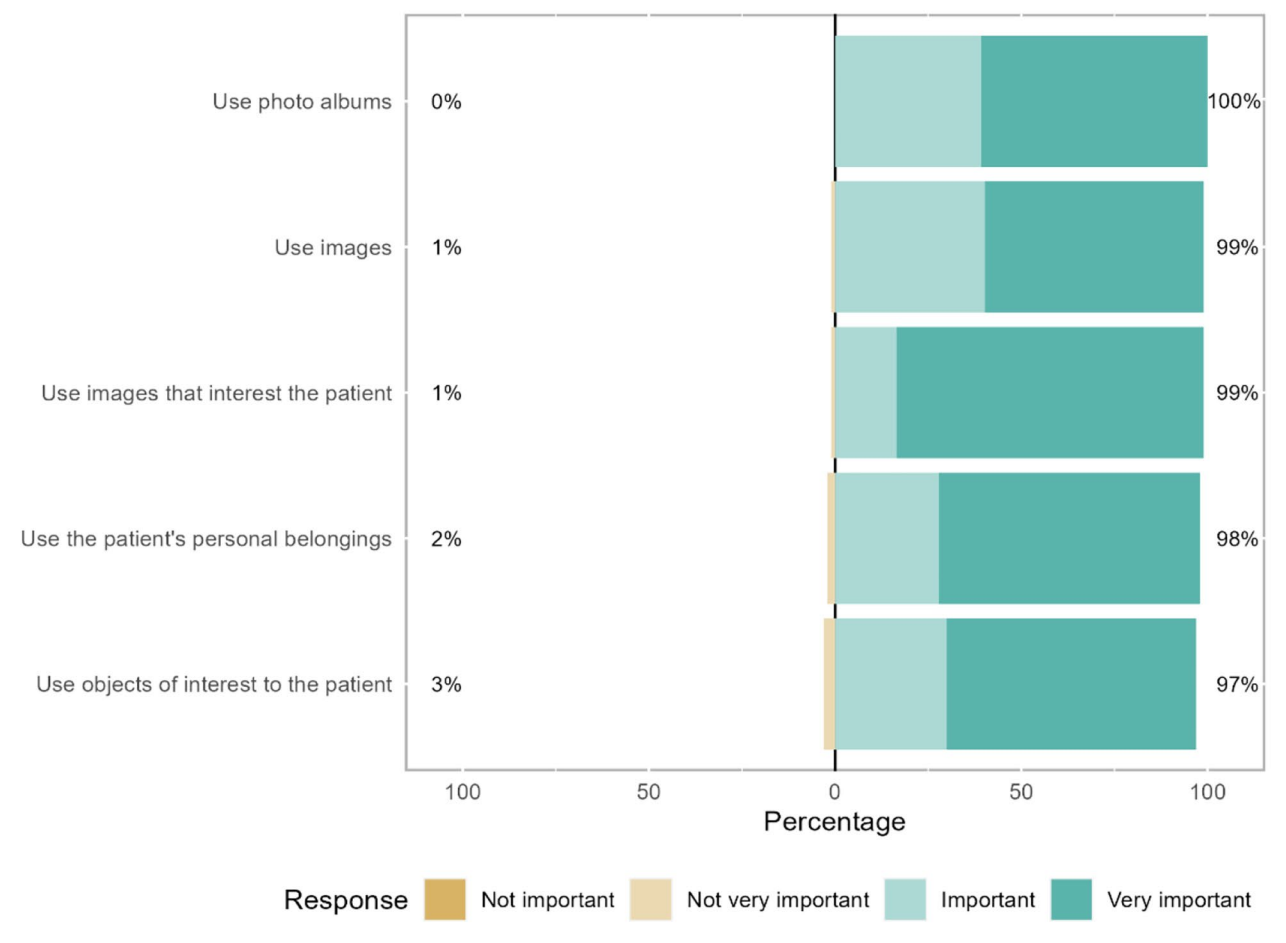
Visual communication strategies

Concerning visual strategies, 83% of therapists consider it “very important” to use images that interest the patient (mean rank = 3.37), while the remaining therapists consider it “important” or “not very important”. The use of personal objects (mean rank = 3.06) is considered “very important” by 70% of the therapists, while the remaining consider it “important” or “not very important” (Fig. 4; Table 2).

**Fig. 5:**
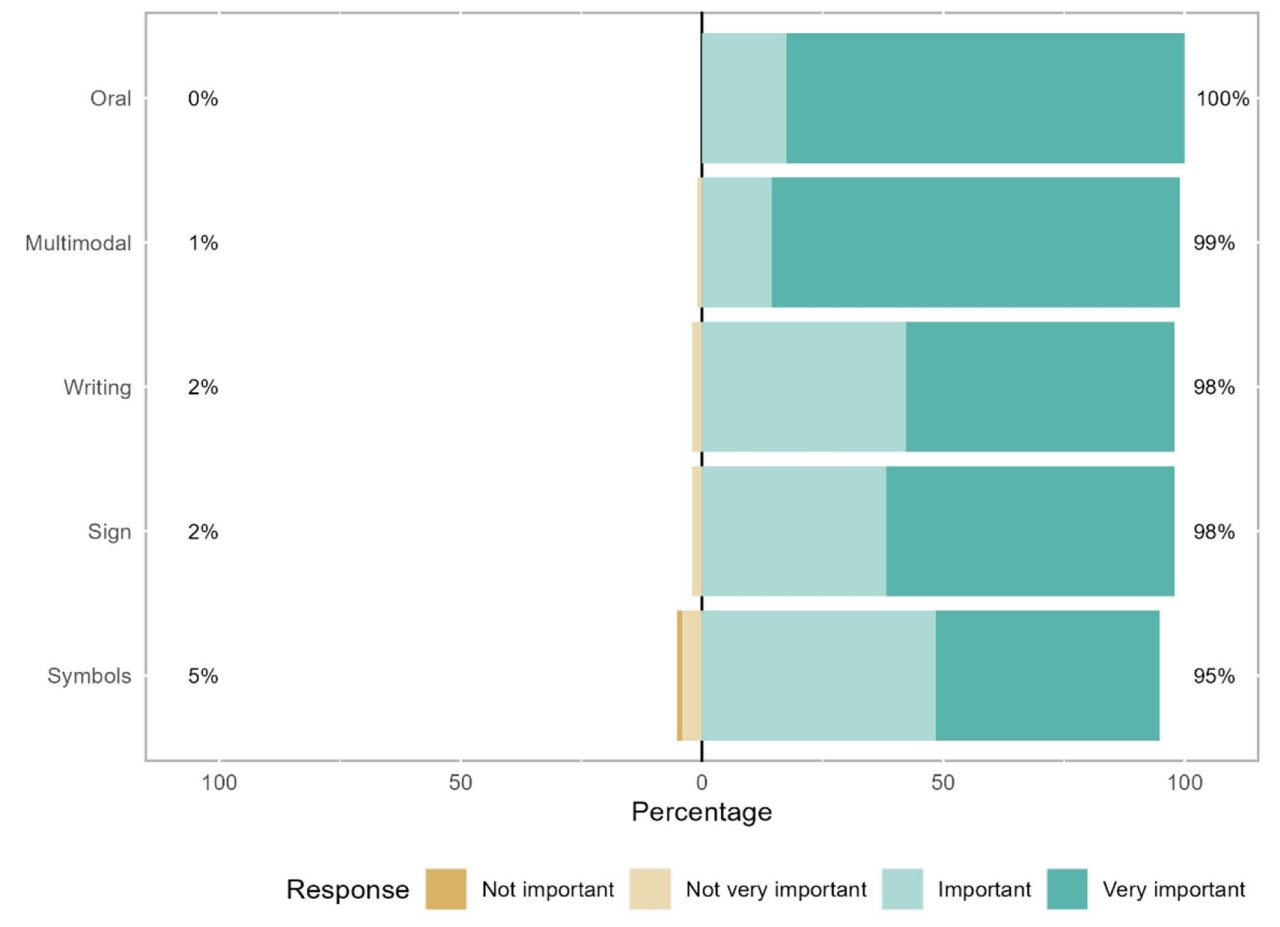
Forms of communication

For 85% of the participants, multimodal communication (mean rank = 3.46) is “very important” and for the remaining participants it is “important” or “not very important”. Additionally, the oral (mean rank = 3.43) form is “very important” for 83% of the participants and “important” for the remaining participants (Fig. 5; Table 2).

**Fig. 6:**
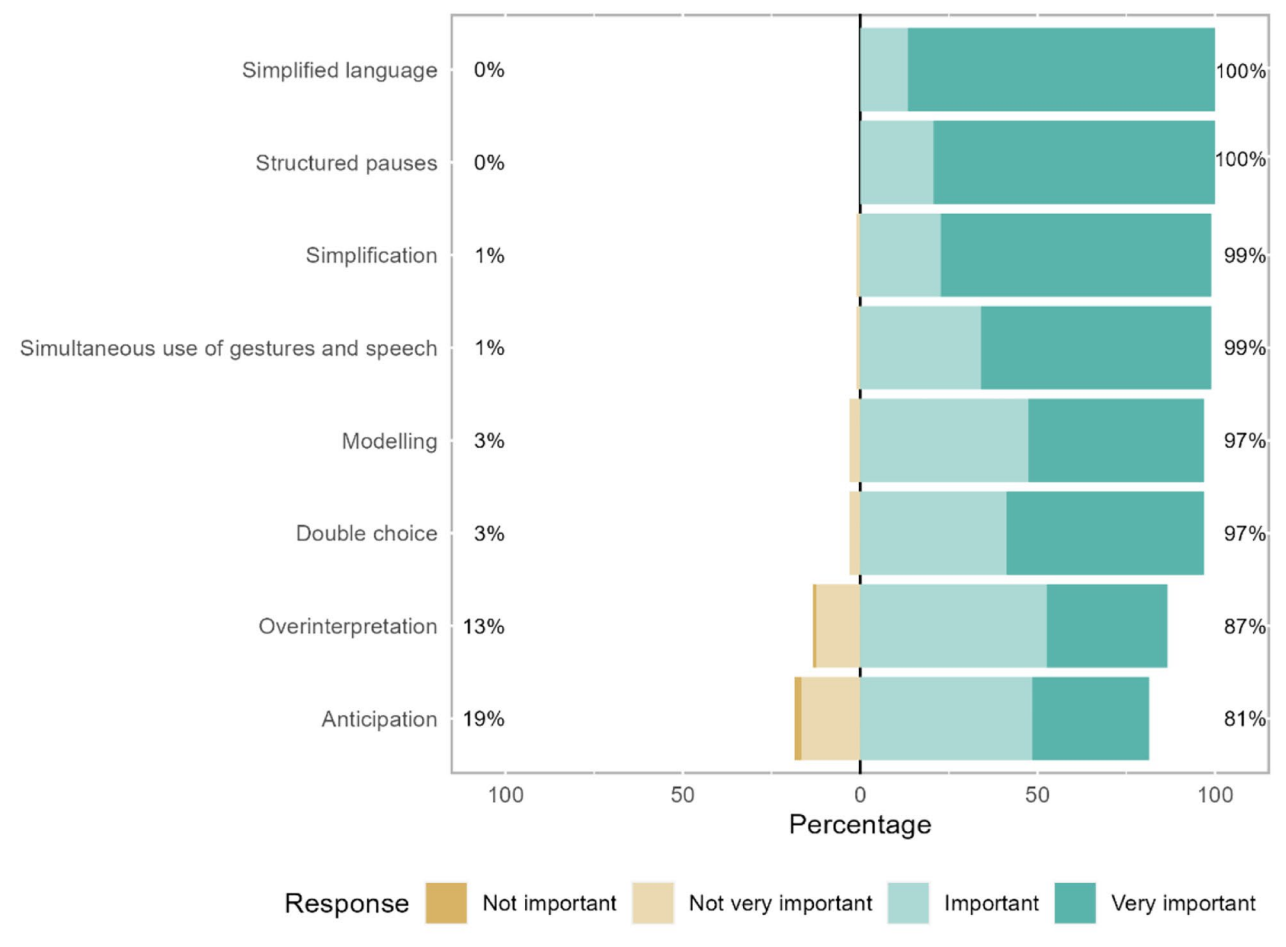
Formal communication strategies

In terms of formal strategies, simplified language (mean rank = 5.62) stands out as “very important” for 87% of respondents and “important” for the remaining respondents. On the other hand, structured pauses (mean rank = 5.34) are emphasised by 79% of respondents as “very important” and “important” for the remaining respondents (Fig. 6; Table 2).

**Fig. 7:**
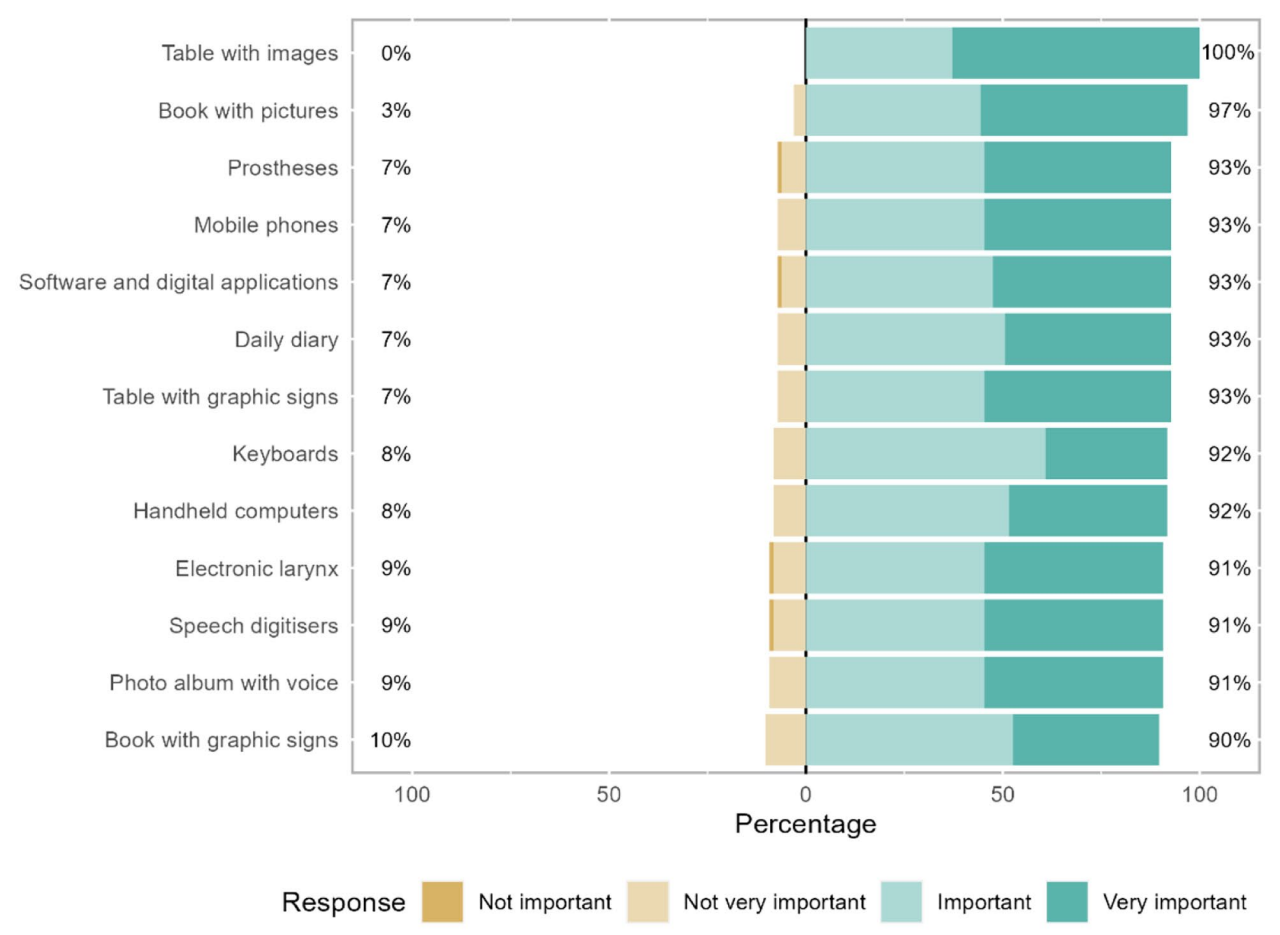
Assistive technologies for communication

Regarding assistive technologies, the strategy of using tables with images (mean rank = 8.39) is most notable, highlighted by 63% of therapists as being “very important” and “important” to the remaining therapists. Picture books (mean rank = 7.62) are considered “very important” by 53% of therapists, while the remaining therapists consider them “important” or “not very important” (Fig. 7; Table 2). Furthermore, although not statistically significant, there was a tendency in the group of therapists with experience in PC to place greater importance on the form of oral communication (*p* = 0.114, Table 2).

**Table 1:** Characterisation of the overall and stratified sample by experience in palliative care

Variable	Total (*n* = 97)	With PC experience (*n* = 40)	Without PC experience (*n* = 57)	***p***-value
Sex, n (%)				0.736
Male	10 (10)	5 (12)	5 (9)	
Female	87 (90)	35 (88)	52 (91)	
Age (years), median (min-max)	27 (22–57)	29 (22–55)	27 (22–57)	0.245
Years of service n (%)				0.875
≤ 5	61 (63)	24 (60)	37 (65)	
6 to 10	23 (24)	10 (25)	13 (23)	
≥11	13 (13)	6 (15)	7 (12)	
Work setting n (%)				
Hospital institution	23 (24)	15 (38)	8 (14)	0.007
Primary healthcare	10 (10)	2 (5)	8 (14)	0.189
Continuing healthcare	27.(28)	21 (53)	6 (11)	< 0.001
Social institutions	18 (19)	4 (10)	14 (25)	0.069
Schools	28 (29)	4 (10)	24 (42)	0.001
Rehabilitation centres	13 (13)	6 (15)	7 (12)	0.699
Retirement homes	13 (13)	10 (25)	3 (5)	0.005
Clinics	27 (28)	8 (20)	19 (33)	0.149
In-home	29 (30)	16 (40)	13 (23)	0.069
Age group worked with n (%)				
Children	83 (86)	28 (70)	55 (97)	< 0.001
Youth	59 (61)	22 (55)	37 (65)	0.325
Adults	65 (67)	38(95)	27(47)	< 0.001

**Legend:** The *p*-value refers to the comparison between groups with and without experience in PC. Work setting and age group variables may apply to more than one category

**Table 2:** Description of the two most important strategies for each group in total and according to whether the participants had experience in palliative care

Variable	Total (n = 97)	With PC experience (n = 40)	Without PC experience (n = 57)	*p*-value
**Environmental**				
Adjusting the patient’s position				0.517
Important	10 (10)	3 (8)	7 (12)	
Very important	87 (90)	37 (93)	50 (88)	
Minimising environmental noise *				0.935
Important	22 (23)	9 (23)	13 (23)	
Very important	74 (77)	31 (78)	43 (77)	
**Non-verbal**				
Establishing eye contact				1.000
Important	5 (5)	2 (5)	3 (5)	
Very important	92 (95)	38 (95)	54 (94)	
Adjusting the pace of speech				0.767
Important	13 (13)	6 (15)	7 (12)	
Very important	84 (87)	34 (85)	50 (88)	
**Verbal**				
Adjusting language level				0.792
Not very important	1 (1)	1 (3)	0 (0)	
Important	5 (5)	2 (5)	3 (5)	
Very important,	91 (94)	37 (93)	54 (95)	
One topic at a time				1.000
Not very important	2 (2)	1 (3)	1 (2)	
Important	7 (7)	3 (8)	4 (7)	
Very important	88 (91)	36 (90)	52 (91)	
**Visuals**				
Images of interest to the patient				1.000
Not very important	1 (1)	0(0)	1 (2)	
Important	16 (17)	7(18)	9 (16)	
Very important	80 (83)	33(83)	47 (83)	
Using patient’s personal belongings				0.829
Not very important	2 (2)	1 (3)	1 (2)	
Important	27 (28)	10 (25)	17 (30)	
Very important	68 (70)	29 (73)	39 (68)	
**Forms of communication**				
Multimodal				0.866
Not very important	1 (1)	0 (0)	1 (2)	
Important	14 (14)	5 (13)	9 (16)	
Very important	82 (85)	35 (88)	47 (83)	
Oral				0.114
Important	17 (18)	4 (10)	13 (23)	
Very important	80 (83)	36(90)	44 (77)	
**Formal strategies**				
Simplified language				0.549
Important	13 (13)	4 (10)	9 (16)	
Very important	84 (87)	36 (90)	48 (84)	
Structured pauses				1.000
Important	20 (21)	8 (20)	12 (21)	
Very important	77 (79)	32 (80)	45 (79)	
**Assistive technologies**				
Table with images				0.523
Important	36 (37)	13 (33)	23 (40)	
Very important	61 (63)	27 (68)	34 (60)	
Book with pictures				0.356
Not very important	3 (3)	0 (0)	3 (5)	
Important	43 (44)	19 (48)	24 (42)	
Very important	51 (53)	21 (53)	30 (53)	

**Legend:** The data is presented as n (%) for each of the levels of importance reported. The *p*-value shown corresponds to the chi-square test or Fisher’s exact test for the comparison between the groups with and without experience in PC

Caption: *One person did not answer this item

## Discussion

To our knowledge, this is the first cross-sectional study to explore the perspectives of SLTs on the most important communication strategies to implement in the context of PC. First, it allowed us to categorise and classify some strategies that these professionals in PC can use. Second, it enabled us to compare the two groups of SLTs and observe that there were no differences in perception in assigning importance to the identified strategies.

Adjusting the patient’s position was the most important in the environmental category. It has been reported that the patient’s position influences the effectiveness of a communication process in the context in which they find themselves [16]. On the one hand, adjusting the patient’s position makes it possible to create empathy with the interlocutors and, on the other, to use some strategies that will allow communication to be carried out in a functional way [16]. Users of augmentative and alternative communication (AAC) systems must be positioned appropriately to operate or manipulate a system [8, 16]. Additionally, the system must be adjusted to the person’s position, whether lying in bed, sitting in an armchair, or standing up [8, 16]. Indeed, an appropriate adjustment to the position provides patients: (i) support, comfort, and availability to use their body according to their mobility; (ii) free movement of the arms and legs, without losing balance (i.e., within their motor skills); (iii) the ability to move freely; and (iv) allows them to maintain the position while using AAC without getting tyred [16]. This strategy could be a fundamental resource for SLTs who use AAC with their patients, helping them to optimise their intervention [17].

Importantly, minimising environmental noise is also critical. Noise is considered an environmental stress factor, and its negative effect on physiological and psychological levels is well known [18]. Specifically, the body reacts to noise in the same way that it reacts to stressful situations [18]. Excessive noise in healthcare settings impairs communication, reducing speech intelligibility and consequently causing the patient tiredness, discomfort, and irritation [18]. For all these reasons, environmental noise is considered a communicative barrier in healthcare since it inhibits the patient’s interaction and communication with healthcare professionals [19]. Additionally, if a person has impaired language comprehension, such as aphasia, auditory and visual distractions may impede their ability to comprehend verbal language [20].

In recent years, studies have demonstrated the significant relevance of environmental strategies in decision-making situations for patients with dementia and aphasia [21–23]. Controlling environmental stimuli, choosing a welcoming setting, reducing environmental noise, and creating quiet environments are some strategies that facilitate communication between the patient and the care provider, as mentioned by various researchers [21–23].

Concerning non-verbal strategies, establishing eye contact was the most important. Eye contact is essential for proximity between the health professional and the patient, who will feel valued and supported [24]. Keutchafo’s [24] studied non-verbal communication between nursing teams and the elderly and revealed that eye contact is often used to gain the patient’s trust and promote a more meaningful interpersonal relationship. A recent study described some communicative strategies that would be useful for patients with communication disorders, solidifying a list of 16 communication strategies [25]. Specifically, the patients reported that communication was facilitated when the professional looked at the patient and emphasised the importance of eye contact during a conversation [25].

Adjusting the pace of speech was another highly relevant strategy for therapists. The importance attributed to this strategy is not recent. Some studies, such as those by Tuohy [26] and Park and Song [27], emphasise that the use of an adjusted pace throughout the speech conveys reassurance and supports the understanding of information [26, 27]. We continue to observe that this strategy remains a highly valued resource. Specifically, in the study mentioned above, participants also reported that speaking clearly while adjusting the pace could facilitate communication [25]. Another study explored what facilitates confidence in communication from the perspective of adults with aphasia and concluded that communicating with partners with an adjusted speech pace helps individuals with aphasia better understand the message [20].

Concerning verbal strategies, the therapists emphasised adjusting to the patient’s language level as “very important”. Clinical language contains specific terminology and abbreviations that the patient is often unaware of [19, 28]. If this is overlooked during an interaction, it could threaten the closeness between the patient and the healthcare professional, as neither will be at the same level of knowledge [19, 28]. Thus, using vocabulary that the patient knows establishes a closer relationship, enabling greater treatment compliance, and a better understanding of the care they will receive [19, 28]. Despite limited research on communication strategies with adults in PC, some studies on decision-making in patients with communication disorders recommend that healthcare professionals use clear language devoid of jargon and passive and complex sentences [29]. Furthermore, individuals with communication disorders who participated in Hickey and colleagues’ research [25] identified that, during a communication situation, interlocutors should use respectful words adjusted to each person’s age [25].

The use of one topic at a time also stood out in the therapists’ perception. Research into communication strategies in the context of advanced dementia has shown that there is a better understanding of information when this strategy is implemented [30]. This result was corroborated by Hickey and colleagues’ study [25], where participants identified giving warnings about changes in conversation topics as an important communication strategy.

Concerning visual strategies, both groups of therapists highlighted employing images and objects of interest to the patient. This group of strategies can include personal photographs, images, personal objects, phrases, and words [31]. In situations of dementia, the use of images and materials of interest to the person are considered facilitating elements for establishing communication [31]. Interventions prioritising this strategy facilitate understanding, promote narratives, stimulate attention, and help patients focus on their health process [31]. Similarly, participants with communication disorders in the study by Hickey and colleagues [25] indicated using images as a helpful communication strategy during clinical situations [25].

A recent scoping review aimed to identify types of decision-making for individuals with aphasia demonstrated that using images to support these individuals’ decisions was only included in two research studies. Using images to support task performance was considered a strategy, but it was used in only one study [32]. Indeed, there is little evidence of the relationship between visual cues and their effectiveness in the decision-making process for patients with communication disorders [31]. However, it has been reported that visual aids improve the decision-making capacity of individuals with dementia in comprehending medical information, employ supportive reasons, and relate this information to their own situation, and contain the potential for judges who majored or are majoring in speech-language pathology to reach a stronger consensus when determining the decision-making capacity of individuals with dementia [31]. Still, it is recognised that utilising these strategies helps healthcare professionals determine, in a safer way, the ability of patients with communication disorders to make their own decisions [31, 33, 34].

The forms of communication are also facilitating strategies. The respondents emphasised multimodal and oral communication as “very important”. Some literature suggests that interventions with patients with aphasia often focus on training non-verbal augmentative communication strategies such as using communication books, computerised systems, gestures, writing, or drawing [35, 36]. Nevertheless, these strategies frequently do not generalise to natural situations [37]. The research by Purdy and Van Dyke [36], although with a very small sample, concluded that eight sessions of multimodal communication training surpassed the use of other approaches [36]. In accordance with our results, studies on people with chronic aphasia advocate the use of multimodal communication since it includes multiple augmentative and alternative resources [38]. Other studies states that this type of communication improves the communicative competence of people with aphasia [38, 39].

Moreover, research shows that oral communication, accompanied by multimodal communication (e.g., images, drawings, gestures, and graphics), promotes comprehension and expression [8, 38]. The participants with communication disorders in Howe and colleagues’ study [20] also indicated that communication becomes easier when there are several alternatives to speech. Notably, while the scientific community recognises the potential of multimodal communication [31], globally, this type of communication has been implemented in multiple contexts (e.g., health and education), as it provides a very consistent form of communication [40]. Nevertheless, there are still many aspects to investigate regarding multimodal communication.

A recent scoping review showed the lack of literature encouraging the use of multimodality by individuals with aphasia concerning decision-making [32]. The importance of gesture use in multimodal communication has also been increasing significantly. Some literature demonstrates that meaning-laden gestures are more likely to attract visual attention than more abstract gestures [37]. Patients with aphasia are more likely to fixate on gestures during speech than healthy participants [37]. Another recent study added the notion that gestures served social and linguistic functions. More specifically, gestures could ratify clinicians’ proxy turns, turn allocation, and turn repair. Additionally, it has been reported that gesture is an effective support for the repair of conversation breakdown typical of persons with language deficits [41]. Furthermore, neuroimaging studies reported a close link at the neural level for the semantic processing of auditory and visual information during communication. Thus, these findings encourage the integration of co-speech gestures during aphasia treatment to improve functional communication for people with aphasia [42].

In terms of formal strategies, simplified language and structured pauses were “very important”. A recent scoping review on decision-making for individuals with aphasia highlights that the strategy of simplified language is significantly referenced in various articles as a facilitator in conveying verbal information to individuals with aphasia, enabling them to make decisions [32]. Others state that using simple language helps the patient understand what is being said, avoiding misunderstandings [28, 43].

The other strategy considered “very important” is structured pause. Like many of the strategies mentioned earlier, we also did not find specific literature on this in the context of PC. However, research on decision-making for individuals with compromised communication has shown that it is essential to make pauses during information transmission [29, 32]. In other studies, patients indicated that, during a conversational situation, it is important for them to have extra time/pauses from clinicians to understand the conveyed message [25]. The same authors also mentioned the need for extra time/pauses to express themselves.

Lastly, in the group of assistive technologies, the strategies considered “very important” had lower percentages than the other categories. Utilising high-tech products that require extensive training may be challenging since brief interventions may be used in PC [2]. However, using low-tech support products is very relevant in PC [2]. Pollens described using several low-tech techniques (e.g., low-tech communication board and written tools for communicating advance directives) to support patients in PC [2, 4, 44]. It is important to point out that there are some preconceived ideas regarding implementing support products [8]. In general, professionals believe that allocating a particular support product depends on a lengthy and time-consuming assessment and is, therefore, not viable for people at the end of life [8]. According to Kelly (2019), quoted by Pollens [2], in PC, evaluations are rarely formal, requiring speed in creating and implementing the different materials.

Nevertheless, AAC in PC increased patients’ quality of life, as they could communicate their needs and wants to their loved ones and the healthcare team [45]. Although the great importance of support products in supported communication is recognised, the costs associated with equipment are often quite high [46]. In addition, generational differences, literacy, and lack of experience with electronic devices can also interfere with the acceptance and implementation of technological equipment [7]. In the case of people with Amyotrophic Lateral Sclerosis, it was reported that some patients prefer to use low-tech products (e.g., tables with images) to communicate with their families [7]. This type of product was considered “very important” by the therapists in this study. For these patients, tools based on AAC are essential for promoting autonomy and improving communication, life quality, and survival [47]. However, it should be emphasised that although support technologies have immense potential for promoting communicative functionality, especially in people with complex communication needs, they must be used in accordance with the particularities of each individual; otherwise, they could present a barrier to participation [48].

Although our study focuses more on the most important strategies to implement in PC, we consider that there is a significant gap in specific literature. Indeed, individuals with aphasia, dementia, or any other pathology compromising communication have the same communicative needs as people receiving PC. Furthermore, the fact that there were no differences between the groups studied is not surprising, since communication strategies can extend across different clinical settings based on the specific characteristics of each person rather than the particular setting.

### Limitations

The size of the sample may limit the results. Specifically, the fact that the sample is rather small may explain the lack of statistically significant differences between SLTs with PC experience and those SLTs without experience in this area. Moreover, although the study was centred on the two strategies given the greatest importance, this does not mean the other strategies are unimportant. Furthermore, there may be other strategies than those presented in the study, so it is recognised that the different groups of strategies may be incomplete.

## Conclusion

In this study, it was observed that verbal and non-verbal strategies were those that were given greater importance by SLTs in the context of communication with adults undergoing PC. There were no differences in perception, in terms of importance, between SLTs with PC experience and SLTs without PC experience, although it was noted that SLTs with experience tend to place greater importance on the form of oral communication.

Through their work, the SLT can implement strategies adapted to different types of communication disorders, helping to personalise and optimise the care provided. It is important that, in the future, more research be conducted on this topic since it may help SLTs improving their clinical interventions in the field of PC and guide the healthcare team in how to best communicate with patients.

### Electronic supplementary material

Below is the link to the electronic supplementary material.

Supplementary Material 1

## Data Availability

The data that support the findings of this study are available on request from the corresponding author.

## Abbreviations

AAC: Augmentative and Alternative Communication
PC: Palliative Care
SLT: Speech-Language Therapist
