# Supplementary material for: Communication strategies for adults in palliative care: the speech-language therapists’ perspective
Source: BMC Palliat Care. 2024 Feb 21;23:49. doi: 10.1186/s12904-024-01382-x (PMC10880300; doi:10.1186/s12904-024-01382-x)
Supplement: Supplementary file 1 — Supplementary Material 1 [file 12904_2024_1382_MOESM1_ESM.docx]

**QUESTIONNAIRE**

**COMMUNICATION STRATEGIES USED BY SPEECH THERAPISTS WITH ADULTS IN PALLIATIVE CARE**

This questionnaire arises from a research project included in the Master's Degree Programme in Palliative Care, at the Faculty of Medicine of the University of Porto.

It aims to identify the communication strategies used with adult patients in Palliative Care, according to their level of importance, based on the analysis of the perceptions of Speech Therapists.

The questionnaire is segmented into three parts. While the first and second sections are designed for all Speech Therapists, the third section is an exception. The final segment is exclusively for Speech Therapists who have experience working with adults in Palliative Care Units or similar settings where adult patients are in palliative care situations. The data collected will be used solely for research purposes, with no financial return.

All the relevant ethical protocols for this type of study were taken into account, prioritising the confidentiality and privacy of the data through the anonymous collection of information.

The completion time for the questionnaire is approximately 10 minutes.

Any questions can be clarified via the following email: [catia.s.o.dias@gmail.com](mailto:catia.s.o.dias@gmail.com).

We thank you in advance for your collaboration.

Please mark with an **X** if you understand the objectives and agree to participate.

After reading the introductory text, I consider myself informed and agree to participate in this study by answering this questionnaire □

I agree for this data to be used for research purposes □

**Section A**

Read the following information carefully and mark your answers with an X.

1. **Sociodemographic Information**
   1. **Sex:** □ Feminine □ Masculine
   2. **Age:** ________ years old

**1.3** **How many years of service do you have:**

□ Up to 5 years

□ From 6 to 10 years

□ From 11 to 15 years

□ From 16 to 20 years

□ From 21-30 years

□ >30 years

**1.4. Workplace** **(You can select more than one option):**

Hospital Institution □ Primary Health Care □ Continuous Care □ Social Institutions □ Elementary Schools and Referral Networks □ Nurseries and Kindergartens □ Rehabilitation Centres □ Nursing homes □ Home care □

**1.5. Which age groups do you support (you can select more than one option):**

Children □ Teenagers □ Adults □

**1.6. ^[[1]](#footnote-1)^Do you work with adult population in palliative situations:**

Yes □ No □

**Section B**

1. **Communication Facilitating Strategies for Adult Patients in Palliative Care**

Read the following information carefully and mark with an X your **perception** of the relevance of each of the strategies, according to the scale presented:

- 1. **What is your perception of the relevance of strategies of the physical space that facilitate communication?**

| **Strategies** | Not important | Slightly important | Important | Very important |
| --- | --- | --- | --- | --- |
| Minimising environmental noise |  |  |  |  |
| Adjusting the patient's position |  |  |  |  |
| Selecting the best physical space |  |  |  |  |
| Adapting the space |  |  |  |  |
| Adjusting brightness |  |  |  |  |

- 1. **What is your perception of the relevance of non-verbal strategies that facilitate communication?**

| **Strategies** | Not important | Slightly important | Important | Very important |
| --- | --- | --- | --- | --- |
| Smile |  |  |  |  |
| Eye contact |  |  |  |  |
| Affective touch |  |  |  |  |
| Physical proximity |  |  |  |  |
| Adjust posture |  |  |  |  |
| Avoid sudden movements |  |  |  |  |
| Inflections in tone of voice |  |  |  |  |
| Speech rhythm |  |  |  |  |
| Vocal intensity |  |  |  |  |
| Vocal segregates (e.g. “humm”, “ah ah…”) |  |  |  |  |
| Vocal qualifiers (e.g. laughter) |  |  |  |  |
| Using silence |  |  |  |  |
| Use universal gestures |  |  |  |  |

- 1. **What is your perception of the relevance of verbal strategies that facilitate communication?**

| **Strategies** | Not important | Slightly important | Important | Very important |
| --- | --- | --- | --- | --- |
| Language level |  |  |  |  |
| Reformulate information |  |  |  |  |
| Repeat information |  |  |  |  |
| Summarise information |  |  |  |  |
| Short sentences |  |  |  |  |
| Address one topic at a time |  |  |  |  |
| Inform of topic change |  |  |  |  |
| Speak slowly |  |  |  |  |
| Ask guiding questions (e.g., “What happened?”; “How do you feel about that?”) |  |  |  |  |
| Use writing |  |  |  |  |
| Write down keywords |  |  |  |  |
| Writing answer options |  |  |  |  |
| Writing down conversation topics |  |  |  |  |
| Write separate topics |  |  |  |  |
| List of words/ phrases frequently used by the patient |  |  |  |  |
| Yes/No answer cards |  |  |  |  |
| Use Portuguese Sign Language gestures |  |  |  |  |

- 1. **What is your perception of the relevance of visual strategies that facilitate communication?**

| **Strategies** | Not important | Slightly important | Important | Very important |
| --- | --- | --- | --- | --- |
| Use photo albums |  |  |  |  |
| Use images that interest the patient |  |  |  |  |
| Use images |  |  |  |  |
| Use objects of interest to the patient |  |  |  |  |
| Use the patient's personal belongings |  |  |  |  |

- 1. **What is your perception of the relevance of facilitating forms of communication?**

| **Forms of communication** | Not important | Slightly important | Important | Very important |
| --- | --- | --- | --- | --- |
| Oral |  |  |  |  |
| Sign |  |  |  |  |
| Written |  |  |  |  |
| Symbols |  |  |  |  |
| Multimodal |  |  |  |  |

- 1. **What is your perception of the relevance of formal communication facilitating strategies?**

| **Strategies** | Not important | Slightly important | Important | Very important |
| --- | --- | --- | --- | --- |
| Simultaneous use of gestures and speech |  |  |  |  |
| Structured pauses |  |  |  |  |
| Overinterpretation |  |  |  |  |
| Double choice |  |  |  |  |
| Simplification |  |  |  |  |
| Simplified language |  |  |  |  |
| Modelling |  |  |  |  |
| Anticipation |  |  |  |  |

- 1. **What is your perception of the relevance of support products that facilitate communication?**

| **Support Products** | Not important | Slightly important | Important | Very important |
| --- | --- | --- | --- | --- |
| Table with graphic signs |  |  |  |  |
| Table with images |  |  |  |  |
| Books with graphic signs |  |  |  |  |
| Books with pictures |  |  |  |  |
| Daily diary |  |  |  |  |
| Photo album with voice |  |  |  |  |
| Handheld computers |  |  |  |  |
| Software and digital applications |  |  |  |  |
| Mobile phones |  |  |  |  |
| Speech digitisers |  |  |  |  |
| Keyboards |  |  |  |  |
| Electronic larynx |  |  |  |  |
| Prostheses |  |  |  |  |

THE QUESTIONNAIRE HAS BEEN COMPLETED

THANK YOU FOR YOUR COLLABORATION

1. Patients who require Palliative Care: advanced stage AIDS patients, patients with so-called advanced organ failures (cardiac, respiratory, hepatic, respiratory, renal), patients with degenerative and severe neurological diseases, patients with dementia in very advanced stage and advanced cancer patients (Portuguese Palliative Care Association - APCP). [↑](#footnote-ref-1)
